# Supplementary figures and images for: Assessing routine health information system performance during the tenth outbreak of Ebola virus disease (2018–2020) in the Democratic Republic of the Congo: A qualitative study in North Kivu
Source: PLOS Glob Public Health. 2022 Jul 29;2(7):e0000429. doi: 10.1371/journal.pgph.0000429 (PMC10021379; doi:10.1371/journal.pgph.0000429)

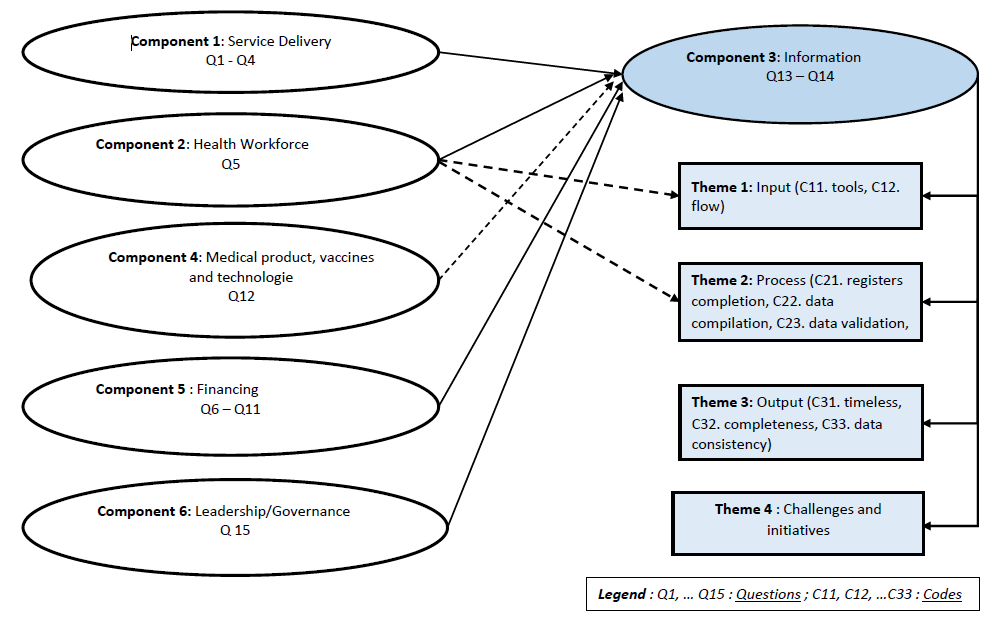

Supplement: S1 Fig — (TIF) [file pgph.0000429.s001.tif]
