# Supplementary material for: Assessing routine health information system performance during the tenth outbreak of Ebola virus disease (2018–2020) in the Democratic Republic of the Congo: A qualitative study in North Kivu
Source: PLOS Glob Public Health. 2022 Jul 29;2(7):e0000429. doi: 10.1371/journal.pgph.0000429 (PMC10021379; doi:10.1371/journal.pgph.0000429)

Author's cover letter for revision

Manuscript Number: PGPH-D-21-01117

Title: Assessing Routine Health Information System Performance during the Tenth Outbreak of Ebola Virus Disease in the Democratic Republic of the Congo: a qualitative study in North Kivu

Authors: Gabriel Kyomba Kalombe, Guillaume Kiyombo Mbela, Serge Manitu Mayaka, Karen A Grépin, Thérèse Mambu Mondo, Celestin Hategeka, Mapatano Mala Ali, Lys Alcayna-Stevens, Serge Kapanga Kule, Joël Konde Nkima-Numbi, Dosithée Ngo Bebe, Pélagie Babakazo Diambalula, Eric Mafuta Musalu, Aimée Lulebo Mampasi, Hinda Ruton, Michael R Law

Date for the revised version: 21th Mars 2022

Authors' responses to reviewer

Authors' responses to reviewer 1

Dear Reviewer #1,

We thank you for your comments. There were no comments that required a response from the authors. Once again, thank you for spending your time to read our manuscript

Authors' responses to reviewer 2

Dear Reviewer #2,

We thank you for your comments and advices how constructive, they help us to improve our manuscripts. Please find below the answers to your observations:

Comments

Title: Consider specifying the year

Answer: the title was changed accordingly (see lines 1-3)

Abstract

Introduction: Consider specifying the year

Answer: the manuscript was changed according to the reviewer's advice (see line 25).

Results: Adversely ? (best to specify in what ways data compilation and validation were affected.)

Answer: the manuscript was changed according to the reviewer's advice (see lines 37,38).

Background

Paragraph/Sentence: Since then, it has undertaken several reforms, including the transition to the District Health Information System (DHIS2) as the online platform for storing, sharing, and analyzing data in its RHIS.

Comment: curious why it's DHIS2. Should this be *second* District Health Information System ?

Answer: the manuscript was changed according to the reviewer's advice (see line 70).

Paragraph/Sentence: For the RHIS to function correctly, tools need to be designed, updated, reproduced, and deployed; training needs to be organized and technologies such as computers and software need to be distributed to all levels of the health system.

Comment: [tools] Such as ? Data entry platforms ?

Answer: the manuscript was changed according to the reviewer's advice (see lines 75,76).

Paragraph/Sentence: Another challenge facing health systems around the world has been the occurrence of major infectious disease outbreaks, which have been shown to have severe consequences on many health system components, including the delivery of health services.

Comment: consider framing this differently, perhaps something along 'The need for effective RHIS is particularly critical during disease outbreaks, when the need for real-time surveillance is…'

Answer: some changes were made in the manuscript according to the reviewer's comments. However, some sentences were maintained in the manuscript as they are related to findings (see line 83-86)

Materials and Methods

Type, site, and period of study

Paragraph/Sentence: The research team hypothesized that the effects of the EVD outbreak were likely to be greater in districts that had recorded the longest duration of the outbreak and/or reported more EVD cases and deaths.

Comment: The authors can just say that they chose districts with high severity to gauze the 'upper bound' of the extent of the effect on RHIS. [putting this as a hypothesis seemed a bit awkward.]

Answer: the manuscript was deeply changed according to the reviewer's advice (see lines 128-132).

Sample Size and KIs selection

Paragraph/Sentence: We achieved saturation with about twenty semi-structured interviews (SSIs). However, we continued interviewing informants until we reached thirty-four KIs

Comment: a) There seems to be a lead sentence missing here; the fact that the authors' sample size determination was based on saturation. b) For quantitative readers, consider explaining what this means.

Answer: some changes were made in the manuscript according to the reviewer's comments, including an explanation of saturation (see lines 160-162)

Paragraph/Sentence: Reasoned choice was also used to select facilities and KIs at different levels.

Question/Comment: what does this [Reasoned choice] mean ?

Answer: We included an explanation of the reasoned choice of key informants (see lines 176-178)

Inclusion and exclusion criteria:

Paragraph/Sentence: Containment measures introduced as part of the fight against Covid-19 limited access to certain structures.

Questions/Comments: So the inclusion criteria for facilities is that they had to be not affected by COVID-19 at the time of the study ? How many facilities were excluded/replaced in the sample from the initial list because of this ?

Answer: some changes were made in the manuscript according to the reviewer's comments. However, the restrictive measures against covid-19 were retained as an barrier to reach some areas that could have

had other realities. No replacement of the targeted structures not visited was carried out as saturation had already been reached (see lines 183-187).

Data collection

Questions/Comments: Nearly all interviews were one-on-one, were conducted at the respondents' place of work, and were recorded.

Questions/Comments: in private ? I recognize it is not always possible to do these interviews in private, but it matters for how accurate the responses are, so consider adding ‘in private’ here if that is true.

Answer: some changes were made in the manuscript according to the reviewer’s comments. We confirm that the vast majority of interviews were conducted in private. We also completed other modalities of conducting interviews that had been deliberately omitted previously to reduce the word count of the manuscript (see lines 192-194).

Results

Number in the table 1. Characteristics of the key informants

Comments: for this journal, I am pretty sure you will need to stick to US stype (i.e., 79.4, not 79,6).

Answer: changes were made by adopting American style (see table 1, lines 146-251).

Footnotes to the table 1

Sentence: Provincial Health Department, including ONG Staff

Comments: Trivial, worth writing the full form here. I wonder if you meant NGO here.

Answer: some changes were made in the footnotes by replacing 'NGO' with 'Non-Governmental Organization (see footnotes under table 1, line 252).

Timeless and completeness

Paragraph/Sentence: Another respondent noted that some private facilities had stopped reporting or were reporting after insistence in protest at the fact they were not covered by financial aids as public facilities. For example, one nurse indicated that:

Comments: [insistence] from whom ?

Answer: We provided more detail on the origin of the insistence on providers to report data (see line 325).

Data consistency or accuracy

Paragraph/Sentence: Some KIs felt that unintentional errors were likely to occur in the templates because of the pressure on providers to send in reports.

Comments: but wasn't this pressure there even before the outbreak ?

Answer: we provided more detail on the context in order to explain insistence on providers to report data during an epidemic. This helped to clarify the difference between this period and the pre Ebola period (see lines 334, 335).

Challenges and initiatives to maintain RHIS optimal functioning

Paragraph/Sentence: The RHIS continued facing several challenges not specific to the EVD outbreak. These include geographical inaccessibility, poor internet coverage, and threats to facilities. Related to the outbreak, some informants felt that facilities were sometimes the victims of attacks because providers were charged to be corrupt and complicit with those who instigated the EVD outbreak for their hidden interests.

Comments: a) Trivial [threats to facilities] From ? b) Minor : for someone who does not know the context, this needs to be clarified.

Answer: some changes were made in the manuscript according to the reviewer's comments. We provided more detail to describe the social and security context in the Ebola-affected area to help readers better understanding the origin of attacks on health facilities by armed groups (see line 343-350).

Paragraph/Sentence: This environment of fear did not facilitate work in general and data reporting activities.

Comment: [did not facilitate] Sounds like it hindered. Why not say that (instead of 'did not facilitate') ?

Answer: some changes were made in the manuscript according to the reviewer's comments (see lines 350, 351).

Paragraph/Sentence: They explained that while workload reduction requires adding staff, fair treatment of agents is likely to stabilize staff who are often in search of the highest bidder.

Comments: a) agents ? b) 'encourage staff to stay in one facility for a longer period' ?

Answer: some changes were made in the manuscript according to the reviewer's comments. Agents has been changed by providers. However, we maintained the idea that better treatment of salaries would allow providers to be more stable and therefore less likely to move from one job to another. This provider stability is recognized as one of the performance factors of health services (see line 399).

Discussion

Paragraph/Sentence: As a result, a measles outbreak was identified late.

Comments: this was not discussed earlier, but is mentioned here as an implication of poor recording/reporting.

Answer: some changes were made in the manuscript according to the reviewer's comments. We have included this result in the section "Data validation and use for decision-making" (see lines 307,308).

Paragraph/Sentence: It is, therefore, possible that decisions that should be taken following the analysis of the data were not taken or were taken late. It is also possible that the decisions taken were not appropriate.

Comments: These are conjectures, and do not follow directly from the analysis of the data the authors collected. Consider removing.

Answer: the manuscript was deeply changed according to the reviewer's advice. The authors' original comment has been removed in the revised version (see lines 436-449).

Paragraph/Sentence: Quantitative studies could help shed light on the magnitude of these data quality concerns. There were no disruptions for some, while there were for others. Among these, the different

activities were affected in the same way while for others, the compilation and holding of data analysis meetings were very much affected.

Comments: a) need to say more here. Or consider removing the sentence. b) From above, it sounds like there was a direct correlation between the magnitude of the effect (Ebola) and RHIS disruption. The authors can mention that here. c) unclear

Answer: the manuscript was deeply changed according to the reviewer's advice. The authors have chosen to say more rather than to delete this idea. We provided more detail on how other quantitative studies can better explore what we did not explore in this study. This includes the interest in assessing the effect of proposed solutions to mitigate the consequences of the epidemic on the functioning of the health information system. We also added details about the relationship between the magnitude of the epidemic and the effects on the functioning of the information system (see lines462,470).

Paragraph/Sentence: In contrast, we did not note the implementation of electronic registers in routine healthcare services despite their advantages.

Comments: [note] Observe, notice ?

Answer: some changes were made in the manuscript according to the reviewer's comments (see line 489).

Paragraph/Sentence: In their systematic review, Wetherill et al (34) noted the inconsistency between the causes of poor data quality and interventions to improve them.

Comments: This paragraph does not jibe well with the rest of the discussion. Not sure how much it adds either.

Answer: The paragraph has been significantly changed. We hope that it is now well linked to the rest of the manuscript (see lines 494-496)

Conclusion

Paragraph/Sentence: The same coordination of the outbreak response and routine activities, the allocation of human resources according to workload, the implementation of a fair and incentive-based salary policy, the simplification and centralization of data flow, the reduction of the number of forms to be completed, and the implementation of electronic registers should be considered to build an efficient RHIS during future public health crises.

<u>Comments</u>: <u>consider rephrasing. 'coordination of the outbreak response and routine activities by the same staff'</u>

Answer: some changes were made in the manuscript according to the reviewer's comments. We have included this result in the section "Data validation and use for decision-making" (see lines 527, 528).

Authors' responses to the Journal requirements:

1. Please ensure that the funders and grant numbers match between the Financial Disclosure field and the Funding Information tab in your submission form. Note that the funders must be provided in the same order in both places as well.

Answer: some changes were made to the section "funding". A clear separation was made between the funding of this research and the funding of the main project from which it emerges. It appears that this study was supported by the personal resources of the principal investigator. Therefore, the changes to this section take into account this detail (see lines 563-571).

2. Please amend your detailed Financial Disclosure statement. This is published with the article, therefore should be completed in full sentences and contain the exact wording you wish to be published. i). State the initials, alongside each funding source, of each author to receive each grant. ii). State what role the funders took in the study. If the funders had no role in your study, please state: "The funders had no role in study design, data collection and analysis, decision to publish, or preparation of the manuscript." iii). If any authors received a salary from any of your funders, please state which authors and which funders.

Answer: Following on from the response to the previous concern and contrary to what we stated previously, no author has received any salary for their participation in this study which emerges from a larger one that has received funding from IRDC. This main project provides a monthly stipend to a doctoral student who developed this research as part of his doctoral dissertation in continuity with the main project but without receiving direct funding from it. The authors who receive salaries as part of the main project were involved in this study to provide their expertise and without any financial benefit. These details justify the changes made to this section (lines 563-571).

3. Please update your Competing Interests statement. If you have no competing interests to declare, please state: "The authors have declared that no competing interests exist."

Answer: the manuscript was changed according to the journal requirements. The declaration of interest has been revised (see line 561).

4. In the online submission form, you indicated that “Data supporting the findings of this study are available from the corresponding author GKK on request.”. All PLOS journals now require all data underlying the findings described in their manuscript to be freely available to other researchers, either 1. In a public repository, 2. Within the manuscript itself, or 3. Uploaded as supplementary information. This policy applies to all data except where public deposition would breach compliance with the protocol approved by your research ethics board. If your data cannot be made publicly available for ethical or legal reasons (e.g., public availability would compromise patient privacy), please explain your reasons by return email and your exemption request will be escalated to the editor for approval. Your exemption request will be handled independently and will not hold up the peer review process, but will need to be resolved should your manuscript be accepted for publication. One of the Editorial team will then be in touch if there are any issues.

Answer: the manuscript was changed according to the journal requirements (see line 584,585). However, the access to data remains assured to researchers upon request to the corresponding author. Indeed, this access should respect the assurances given to the ethical committee of the Kinshasa School of Public Health. In fact, even if the study key informants had been informed about sharing data with other researchers, this access should not in any case concern information that could allow the identification of respondents.

5. Please provide separate figure files in .tif or .eps format only and ensure that all files are under our size limit of 20MB.

Answer: the graphics files have been converted to the required format (.tif) and respect the 20 MB size limit

Additional Editor Comments (if provided): The authors should follow COREQ guideline for qualitative research reporting. In the guideline, the authors should provide overall coding tree and give examples of coding process. The authors also should provide explanation on researchers subjectivity and reflexivity (see COREQ). In addition to that, please respond to reviewers comment and give revisions accordingly.

Answer: The authors evaluated the manuscript on the basis of the COREQ criteria for qualitative studies. The pages of the manuscript where the reader can find the different criteria of the COREQ have been

filled in. Criteria that are not applicable have been marked N/A. The COREQ check-list is attached to the revised version of the manuscript. In line with these criteria, a section has been inserted in the manuscript to provide information on the gender, occupation and experience of the authors (see lines 556-558), as well as the quality of the principal investigator (see line 191).or other authors' contributions related to coding (see line 558).

Sincerely,

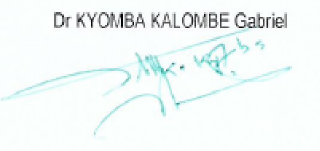

Supplement: Attachment — Submitted filename: Response to Reviewers.pdf [file pgph.0000429.s002.pdf]
